# Supplementary material for: SUMOylation of Jun fine-tunes the Drosophila gut immune response
Source: PLoS Pathog. 2022 Mar 7;18(3):e1010356. doi: 10.1371/journal.ppat.1010356 (PMC8929699; doi:10.1371/journal.ppat.1010356)
Supplement: S1 Fig — (PDF) [file ppat.1010356.s001.pdf]

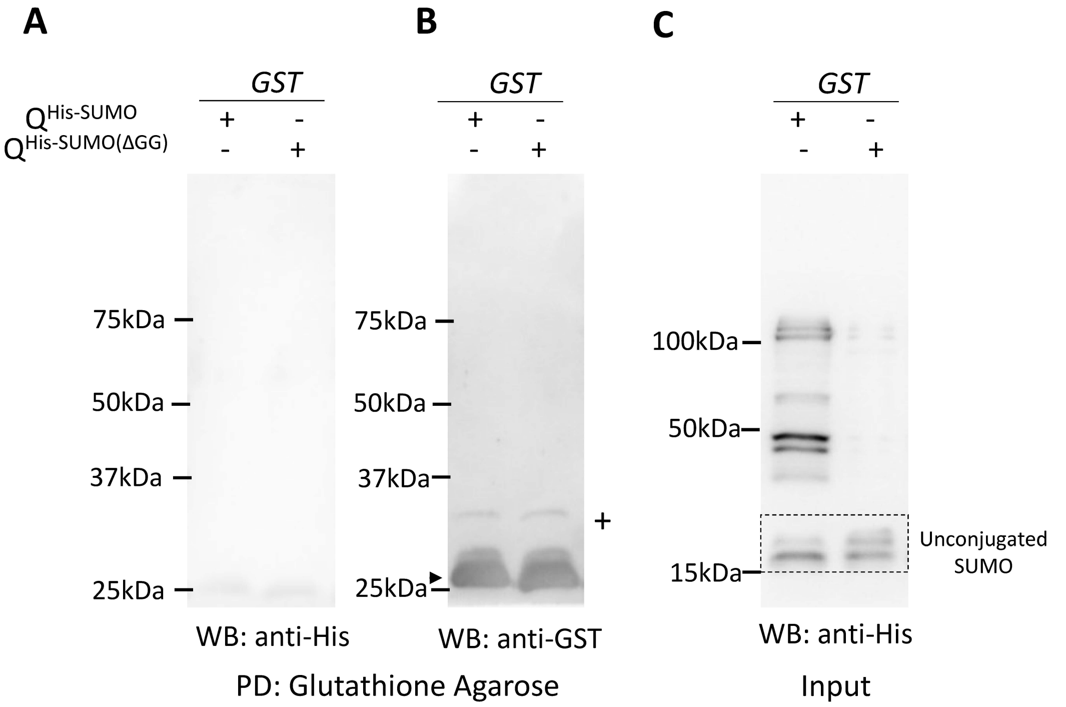

Figure S1: GST is not SUMO conjugated *in-bacto*.

GST has earlier been shown not to be SUMO-conjugated (Fig 1C, Nie et. al., 2009), by the Courey lab, which developed the *in-bacto* SUMO conjugation system utilized in our study.

A. In our hands, we have reproduced the result with Anti-His western blot (left panel) post Glutathione agarose affinity pulldown (PD) showing no SUMOylated bands for GST. Here, GST co-transformed with non-conjugatable *SUMO (SUMO-ΔGG)* serves as a negative control for SUMO conjugation. SUMOylated band is expected around the 37 kD m.w. marker.

B. Anti-GST WB (middle panel) post affinity PD indicates similar levels of GST for the experimental and control conditions. The '+' marks a non-specific band, SUMOylated band is expected around the 37 kD m.w. marker.

C. Anti-His WB (right panel) of the lysates prior to PD shows free SUMO/unconjugated SUMO expressing in comparable levels in SUMO-GG and SUMO-ΔGG. The higher molecular weight bands seen in SUMO-GG lane could be conjugation of *Drosophila* SUMO to bacterial proteins.

Reference

1. Nie M, Xie Y, Loo JA, Courey AJ. Genetic and proteomic evidence for roles of Drosophila SUMO in cell cycle control, Ras signaling, and early pattern formation. PLoS One. 2009. Jun 16;4(6):e5905. doi: 10.1371/journal.pone.0005905.
