# Supplementary material for: SUMOylation of Jun fine-tunes the Drosophila gut immune response
Source: PLoS Pathog. 2022 Mar 7;18(3):e1010356. doi: 10.1371/journal.ppat.1010356 (PMC8929699; doi:10.1371/journal.ppat.1010356)
Supplement: S2 Fig — (PDF) [file ppat.1010356.s002.pdf]

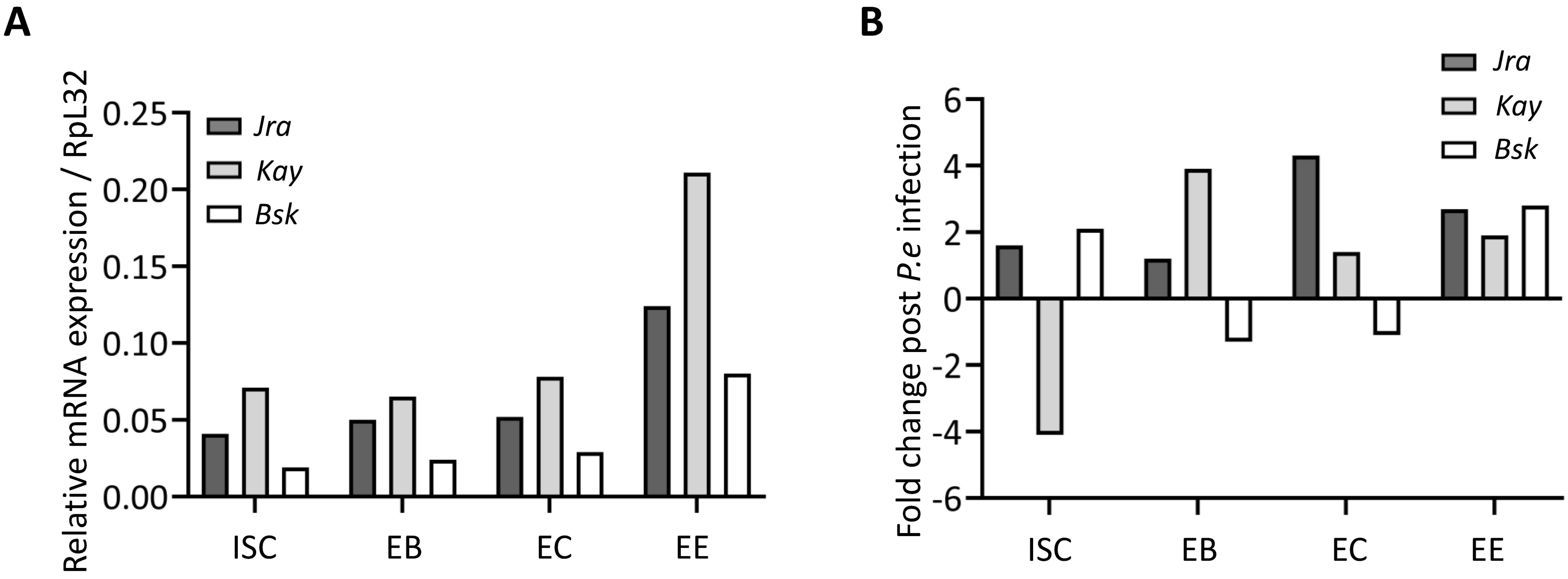

Figure S2: Expression of JNK components in different cell types of the adult gut.

A. Relative mRNA expression of JNK pathway components in different cell types of the adult gut. ISC, Interstitial stem cells; EB, Enteroblast; EC, Enterocytes; EE, Enteroendocrine cells.

B. Fold change of Bsk, Jra and Kay post oral infection with *P.e.*

Graphs plotted from data available at http://flygutseq.buchonlab.com/
