# Supplementary material for: SUMOylation of Jun fine-tunes the Drosophila gut immune response
Source: PLoS Pathog. 2022 Mar 7;18(3):e1010356. doi: 10.1371/journal.ppat.1010356 (PMC8929699; doi:10.1371/journal.ppat.1010356)
Supplement: S3 Fig — (PDF) [file ppat.1010356.s003.pdf]

A

Figure 2	No of Flies used for the experiment	# experiments
A	w^{1118} (UC) – 84; w^{1118} (I) – 154; Jra^{IA109}/+ (UC) – 83; Jra^{IA109}/+ (I) – 161; $Jra^{76\text{-}19}$/+ (UC) – 87; $Jra^{76\text{-}19}$/+ (I) – 141.	3
B	>w^{1118} (C) – 103; >w^{1118} (I) – 141; >Jra^{RNAi} (C) – 91; >Jra^{RNAi} (I) – 141; >Jra^{DN} (C) – 103; >Jra^{DN} (I) – 159.	3
C	>w^{1118} (UC) – 66; >w^{1118} (I) – 77; >Bsk^{DN} (UC) – 79; >Bsk^{DN} (I) – 89 >Kay^{DN} (UC) – 102; >Kay^{DN} (I) – 89.	2
D	>w^{1118} (UC) – 86; >w^{1118} (I) – 128; >$SUMO^{RNAi}$ (UC) – 91; >$SUMO^{RNAi}$ (I) – 141.	3
E	>w^{1118} (C) – 20; >w^{1118} (I) –51; >$Uba2^{RNAi}$ (C) – 18; >$Uba2^{RNAi}$ (I) – 50; >Aos^{RNAi} (C) – 17; >Aos^{RNAi} (I) – 47; >$Ubc9^{DN}$ (C) – 17; >$Ubc9^{DN}$ (I) – 24.	1
F	w^{1118} (2 hpi) – 20; Jra^{IA109}/+ (2 hpi) – 20; w^{1118} (8 hpi) – 17; Jra^{IA109}/+ (8 hpi) – 19; w^{1118} (24 hpi) – 20 ; Jra^{IA109}/+ (24 hpi) – 19.	2

B

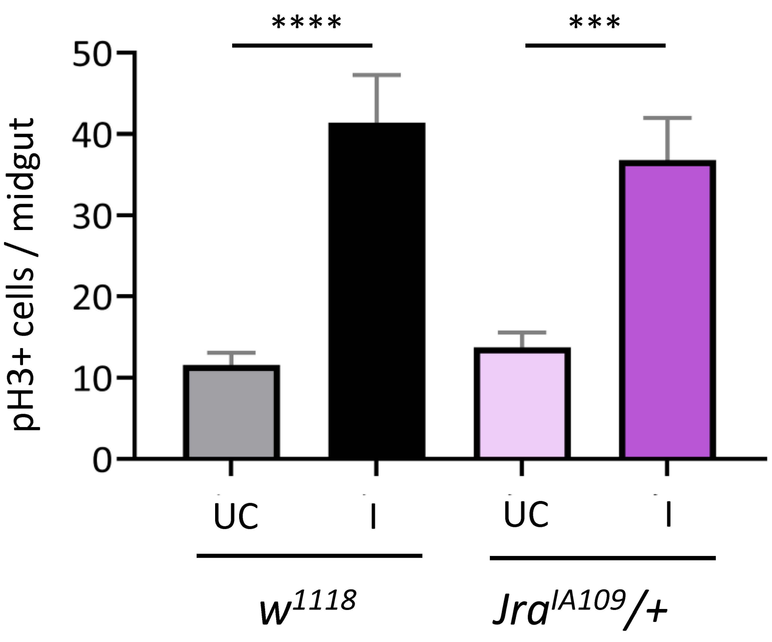

Figure S3: Jra regulates the gut immune response.

A. Tabular representation of the total number of flies used for experiments and number of independent experiments performed in Fig 2.

B. Quantitation of the number of $pH3^+$ cells per midgut of UC flies and flies orally fed with *P.e* ****$p<0.0001$; ***$p=0.0011$ as determined by 2-way ANOVA with Tukey's post-hoc test. Number of gut used; w^{1118} (UC) – 16; w^{1118} (I) – 17; Jra^{IA109}/+ (UC) – 14; Jra^{IA109}/+ (I) – 17. Means and SEMs represented.
