# Supplementary material for: SUMOylation of Jun fine-tunes the Drosophila gut immune response
Source: PLoS Pathog. 2022 Mar 7;18(3):e1010356. doi: 10.1371/journal.ppat.1010356 (PMC8929699; doi:10.1371/journal.ppat.1010356)
Supplement: S4 Fig — (PDF) [file ppat.1010356.s004.pdf]

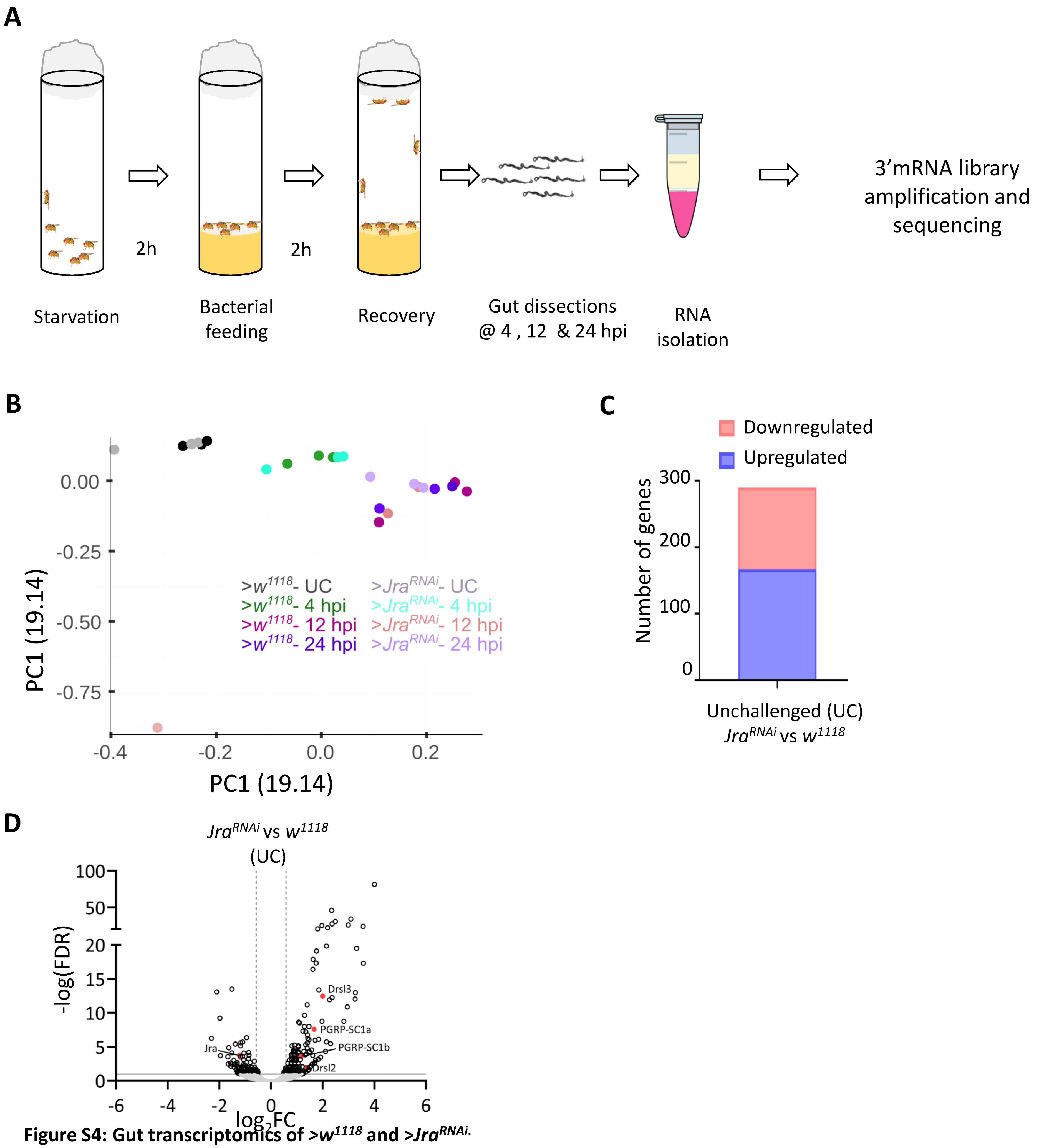

Figure S4: Gut transcriptomics of >*w*[1118] and >*Jra*[RNAi].

A. Schematic of 3' mRNA sequencing experiment. Flies were initially starved (without food and water) for 2h. Flies were then transferred onto a filter paper soaked in concentrated culture of *P.e* and 5% sucrose solution. 2h post feeding, flies were transferred to normal fly food and guts were dissected at 4, 12 and 24 hours post infection (hpi). Gut tissue was homogenized in trizol and RNA was extracted for 3'mRNA sequencing.

B. Principal component analysis (PCA) plot showing the first two principal components of the entire data set.

C. Bar plot representation of the total number of significantly differentially expressed genes (FDR<0.1) obtained at different hpi after comparing to UC animals of the respective genotype.

D. Volcano plot showing the up or down-regulated genes, comparing *NP-Gal4*[ts] >*w*[1118] and *NP-Gal4*[ts] >*Jra*[RNAi]. A few genes previously known to function in regulating immunity are highlighted.
