# Supplementary material for: SUMOylation of Jun fine-tunes the Drosophila gut immune response
Source: PLoS Pathog. 2022 Mar 7;18(3):e1010356. doi: 10.1371/journal.ppat.1010356 (PMC8929699; doi:10.1371/journal.ppat.1010356)
Supplement: S5 Fig — (PDF) [file ppat.1010356.s005.pdf]

A

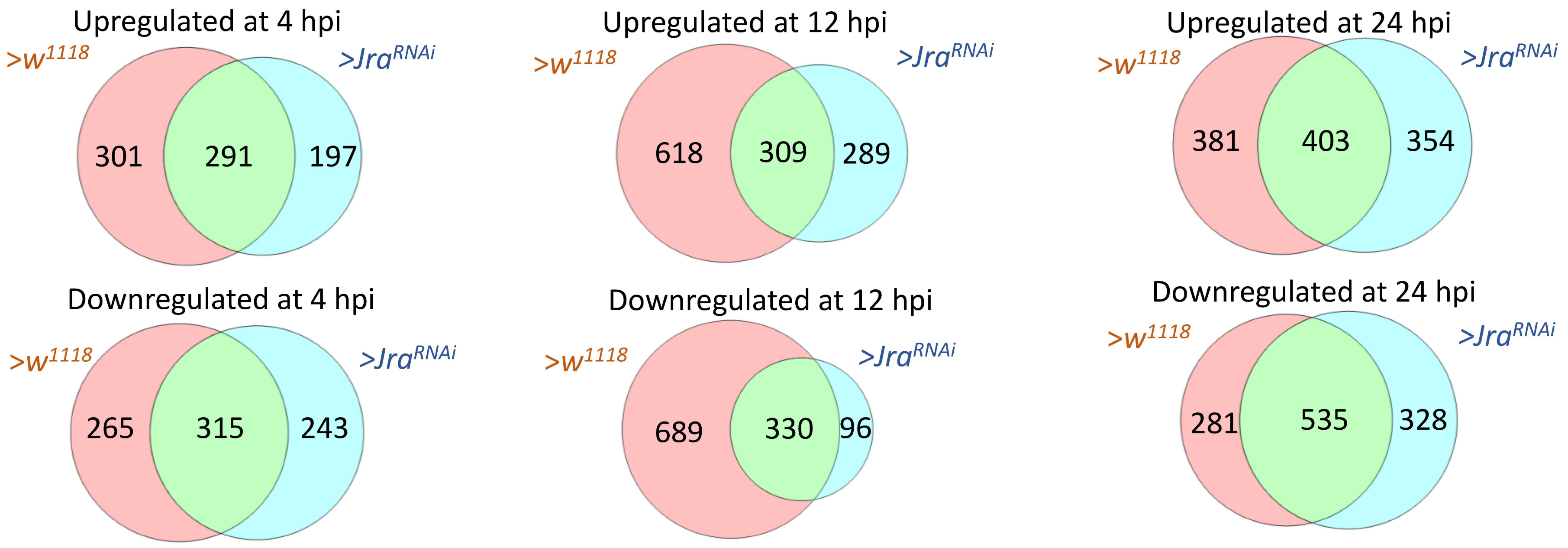

B

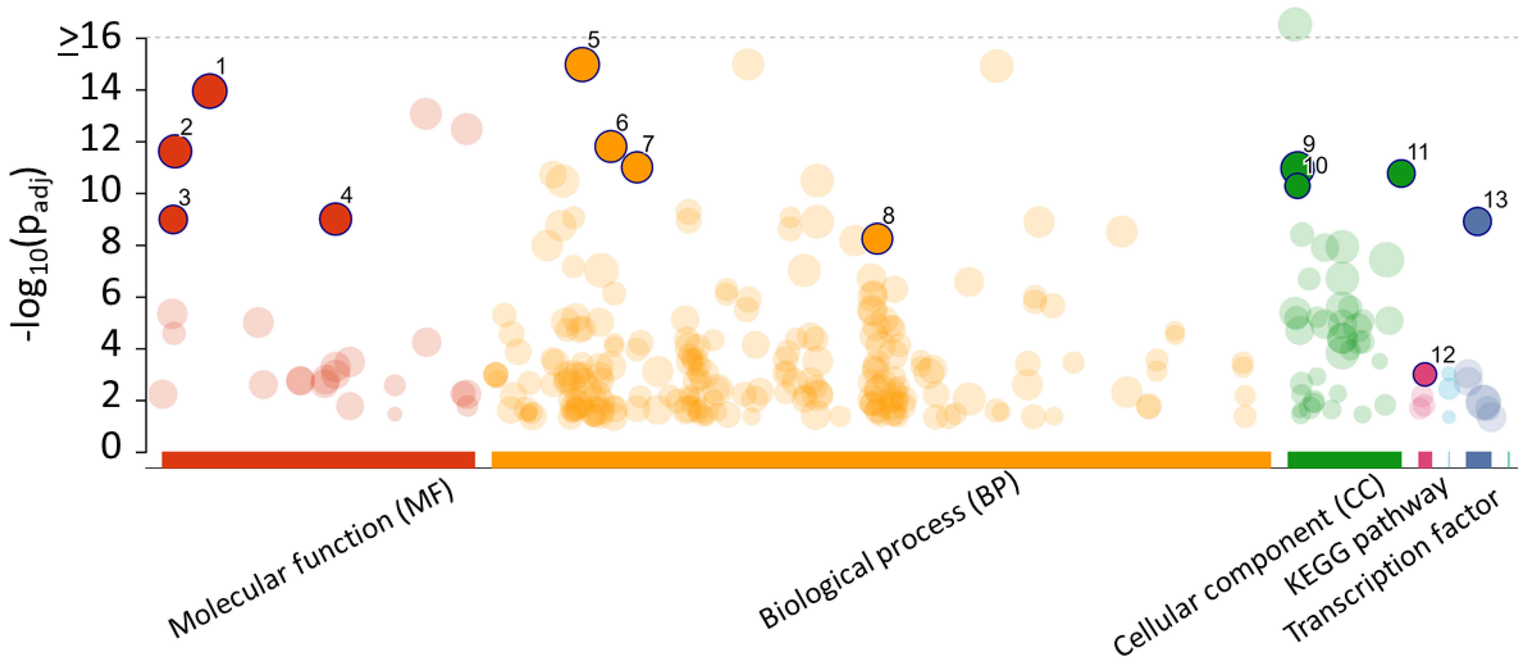

#	GO term
1	Binding
2	Catalytic activity
3	RNA binding
4	Ion binding
5	Metabolic process
6	Gene expression
7	RNA metabolic process
8	System development
9	Cytoplasm
10	Nucleolus
11	Ribonucleoprotein complex
12	Endocytosis
13	GATAe

Figure S5: Comparison of Gut transcriptomics of >*w*^{1118} and >*Jra*^{RNAi} *in terms of overlap and Gene Ontology.*

A. Venn diagram of significantly differentially expressed genes (FDR<0.1) upregulated and downregulated in >*w*^{1118} and >*Jra*^{RNAi}. The intersection shown in a shade of green represent genes that are significantly differentially expressed in >*w*^{1118} and >*Jra*^{RNAi}.

B. Gene ontology (GO) enrichment analysis of the significantly differentially expressed genes in >*w*^{1118} and >*Jra*^{RNAi} at 4, 12 and 24 hpi. Some key GO terms are highlighted and described
