# Supplementary material for: SUMOylation of Jun fine-tunes the Drosophila gut immune response
Source: PLoS Pathog. 2022 Mar 7;18(3):e1010356. doi: 10.1371/journal.ppat.1010356 (PMC8929699; doi:10.1371/journal.ppat.1010356)
Supplement: S6 Fig — (PDF) [file ppat.1010356.s006.pdf]

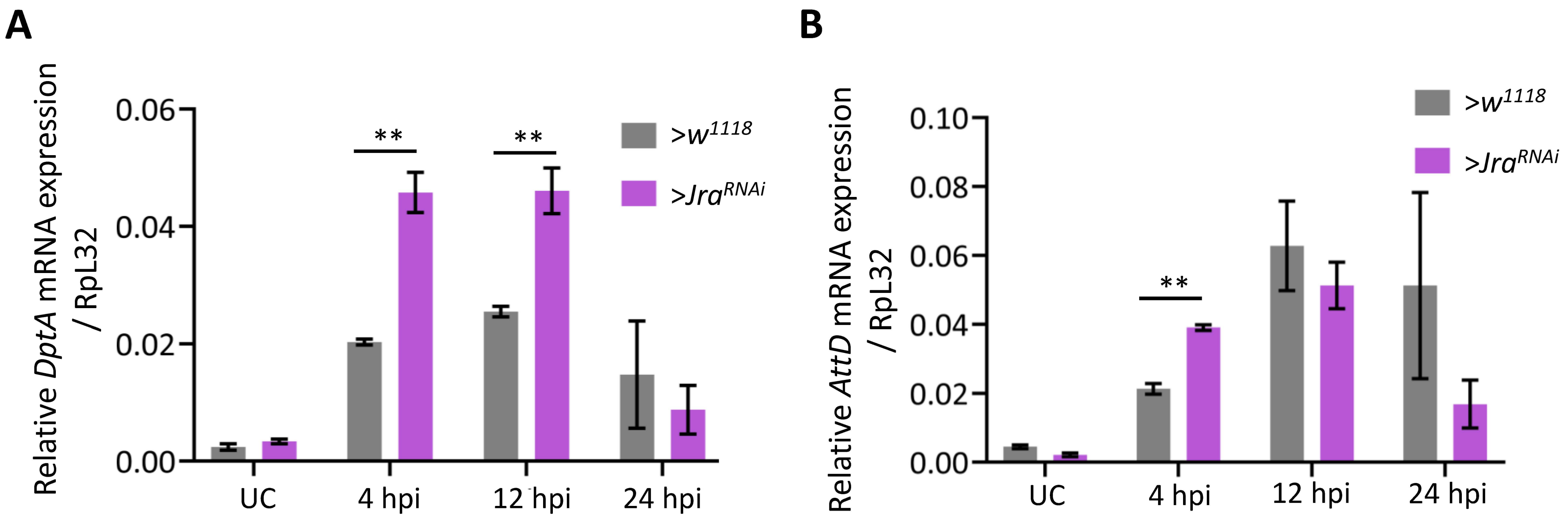


**Figure S6: >*Jra*$^{RNAi}$ shows increased expression of AMPs during infection.**
qRT-PCR data showing the activation of *DptA* (**A**) and *AttD* (**B**) transcripts during gut infection in >*w*$^{1118}$ and >*Jra*$^{RNAi}$. **p<0.0091 as determined by 2-way ANOVA with Bonferroni's post-hoc test for multiple comparisons. Data from three independent experiments. Means and SEMs represented.
