# Supplementary material for: SUMOylation of Jun fine-tunes the Drosophila gut immune response
Source: PLoS Pathog. 2022 Mar 7;18(3):e1010356. doi: 10.1371/journal.ppat.1010356 (PMC8929699; doi:10.1371/journal.ppat.1010356)
Supplement: S7 Fig — (PDF) [file ppat.1010356.s007.pdf]

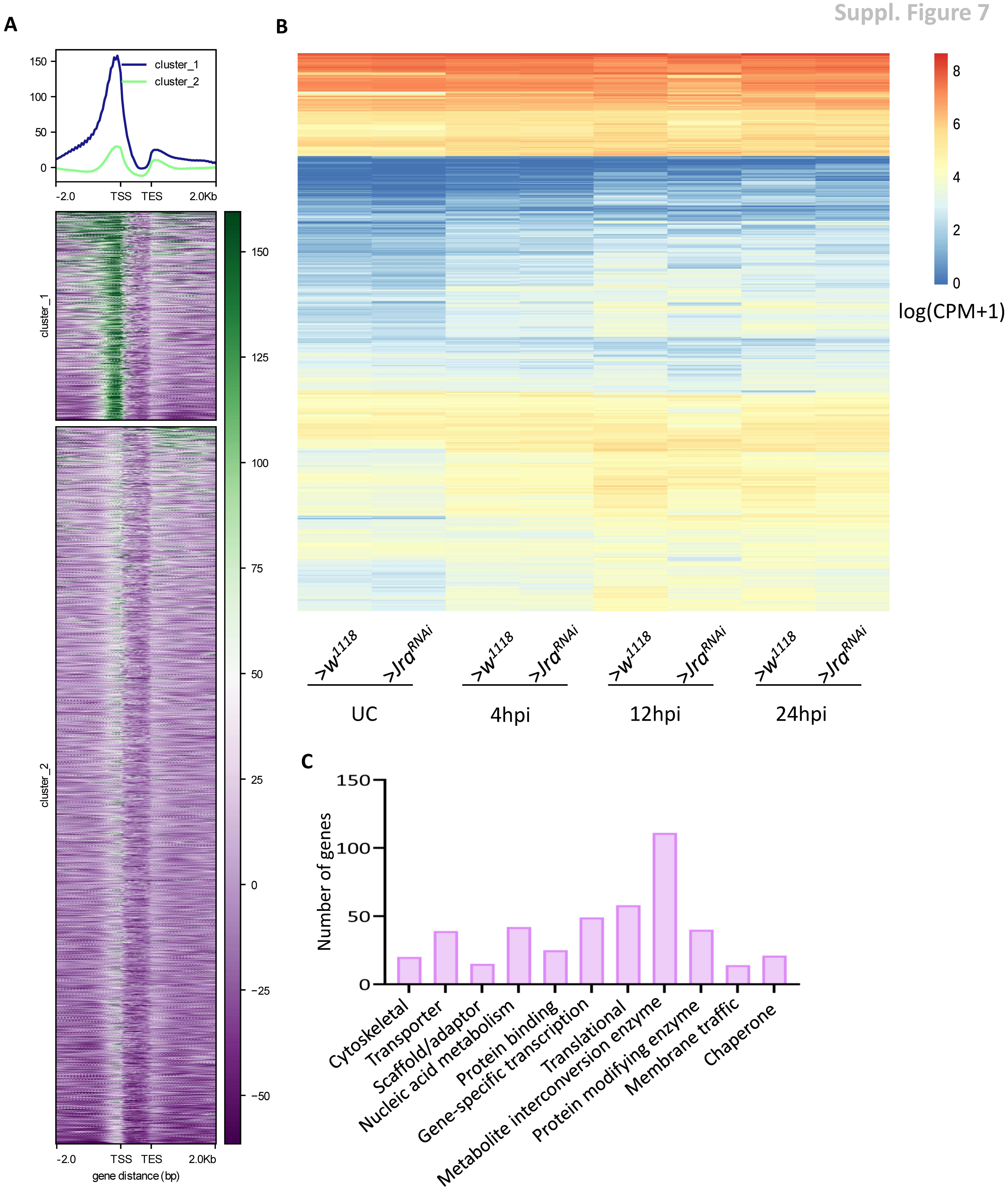

Figure S7: Mapping Jra occupancy on the promoters of differentially expressed genes in >*w*^{1118} and >*Jra*^{RNAi}.

A. Heatmap showing occupancy of Jra on the gene body of the differentially expressed genes in >*w*^{1118} and >Jra^{RNAi}. Cluster 1 represents a set of genes with enriched binding. Heatmap plotted with data extracted from Jra ChIP-seq dataset (ENCSR471GSA) post input (ENCSR908EFA) normalization

B. Heatmap representing the normalised expression counts of genes with enriched Jra (Cluster 1) binding on the promoters.

C. GO terms (Panther-Protein class) of genes with enriched Jra binding on the promoters.
