# Supplementary material for: SUMOylation of Jun fine-tunes the Drosophila gut immune response
Source: PLoS Pathog. 2022 Mar 7;18(3):e1010356. doi: 10.1371/journal.ppat.1010356 (PMC8929699; doi:10.1371/journal.ppat.1010356)
Supplement: S8 Fig — (PDF) [file ppat.1010356.s008.pdf]

Suppl. Figure 8

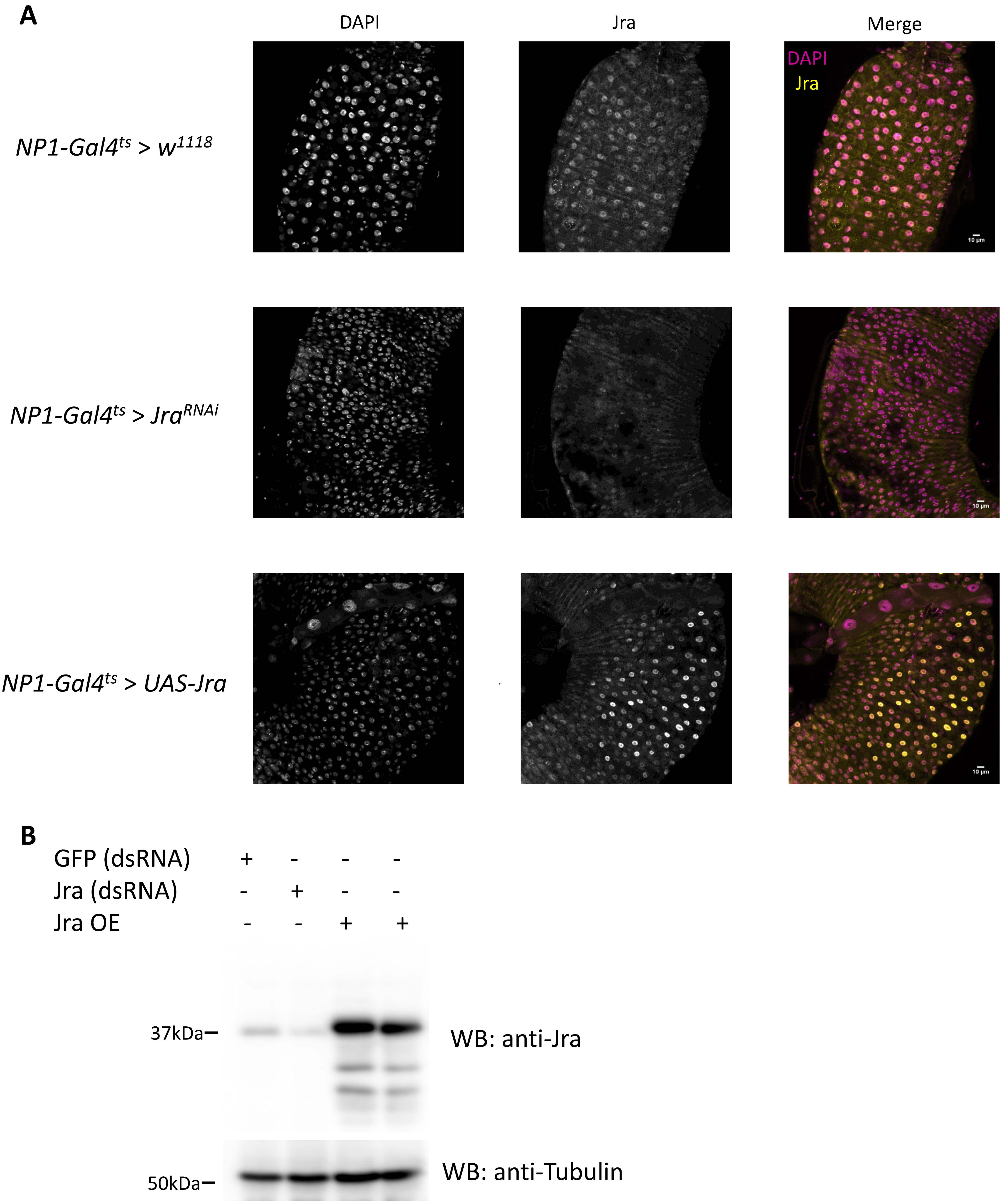

Figure S8: Validation of Jra antibody.

A. Immunofluroscence (IF) images showing Jra specific staining (yellow) in the gut tissues of different genotypes. *UAS-Jra*^{RNAi} (middle panel) and *UAS-Jra* (bottom panel) show contrasting results as compared to the control (top panel). Purified Jra antibody was used for the experiment.

B. To test the specificity of Jra antibody, 529SU cells were treated with 10ug of GFP dsRNA (control) and Jra dsRNA. In addition, cells were transiently transfected with pRM-Jra^{WT} (Jra OE, lane 3) and pRM-Jra^{SCR} (Jra OE, lane 4) to overexpress Jra. WB for anti-Jra was performed using purified Jra specific antibody. Anti-Tubulin WB was used as a loading control.
