# Supplementary material for: SUMOylation of Jun fine-tunes the Drosophila gut immune response
Source: PLoS Pathog. 2022 Mar 7;18(3):e1010356. doi: 10.1371/journal.ppat.1010356 (PMC8929699; doi:10.1371/journal.ppat.1010356)
Supplement: S9 Fig — (PDF) [file ppat.1010356.s009.pdf]

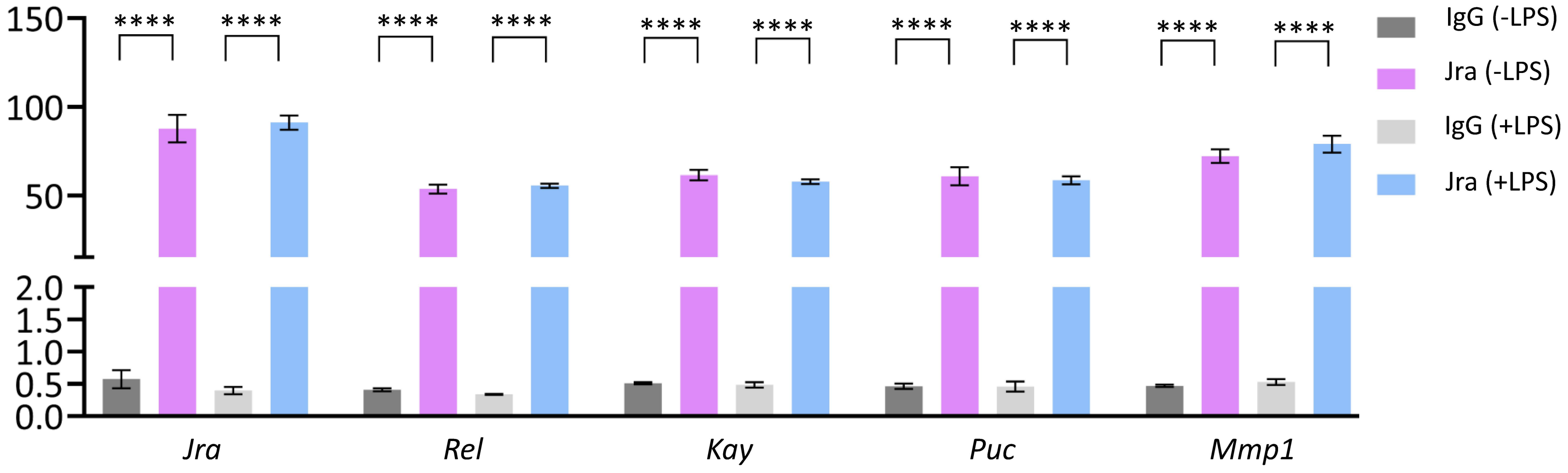

Figure S9: Jra regulates the transcription by directly binding to the promoters of key factors.
qRT-PCR of Jra specific ChIP (performed in 529SU cells) showing occupancy on the promoters of *Jra*, *Rel*, *Kay*, *Puc* and *Mmp1.* Enrichment seen over IgG for each condition/gene. Oneway Anova was used for pairwise comparisons. ****p<0.001. Data represented from there independent experiments. Mean and SEM represented.
