# Supplementary material for: SUMOylation of Jun fine-tunes the Drosophila gut immune response
Source: PLoS Pathog. 2022 Mar 7;18(3):e1010356. doi: 10.1371/journal.ppat.1010356 (PMC8929699; doi:10.1371/journal.ppat.1010356)
Supplement: S10 Fig — (PDF) [file ppat.1010356.s010.pdf]

A

Figure 5	No of Flies used in the experiment	# experiments
A	Jra^{WT} (UC) – 142; Jra^{WT} (I) – 154; Jra^{SCR} *L1* (UC) – 103; Jra^{SCR} *L1* (I) – 154; Jra^{SCR} *L2* (UC) – 111; Jra^{SCR} *L2* (I) – 127.	3
B	Jra^{WT} (UC) – 51; Jra^{WT} (I) – 99; Jra^{SCR} (UC) – 49; Jra^{SCR} (I) – 74; Jra^{IA109}/Jra^{WT} (UC) – 41; Jra^{IA109}/Jra^{WT} (I) – 95; Jra^{IA109}/Jra^{SCR} (UC) – 46; Jra^{IA109}/Jra^{SCR} (I) – 92.	2
C	Jra^{SCR}, *NP1-Gal4*^{ts}/Jra^{SCR} (UC) – 84; Jra^{SCR}, *NP1-Gal4*^{ts}/Jra^{SCR} (I) – 168; Jra^{SCR},*NP1-Gal4*^{ts}/Jra^{WT} (UC) – 89; Jra^{SCR},*NP1-Gal4*^{ts}/Jra^{WT} (I) – 185; Jra^{SCR},*NP1-Gal4*^{ts}/Jra^{SCR};*UAS-*Jra^{WT}/+ (UC) – 80; Jra^{SCR},*NP1-Gal4*^{ts}/Jra^{SCR};*UAS-*Jra^{WT}/+ (I) – 162; Jra^{SCR},*NP1-Gal4*^{ts}/Jra^{SCR};*UAS-*Jra^{SCR}/+ (UC) – 82; Jra^{SCR},*NP1-Gal4*^{ts}/Jra^{SCR};*UAS-*Jra^{SCR}/+ (I) – 163.	3
D	Jra^{SCR};*Jra-Gal4/+* (UC) – 142; Jra^{SCR};*Jra-Gal4/+* (I) – 155; Jra^{SCR}/Jra^{WT};*Jra-Gal4/+* (UC) – 117; Jra^{SCR}/Jra^{WT};*Jra-Gal4/+* (I) – 153; Jra^{SCR};*Jra-Gal4/UAS-*Jra^{WT} (UC) – 136; Jra^{SCR};*Jra-Gal4/UAS-*Jra^{WT} (I) – 155; Jra^{SCR};*Jra-Gal4/UAS-*Jra^{SCR} (UC) – 132; Jra^{SCR};*Jra-Gal4/UAS-*Jra^{SCR} (I) – 150.	3

B

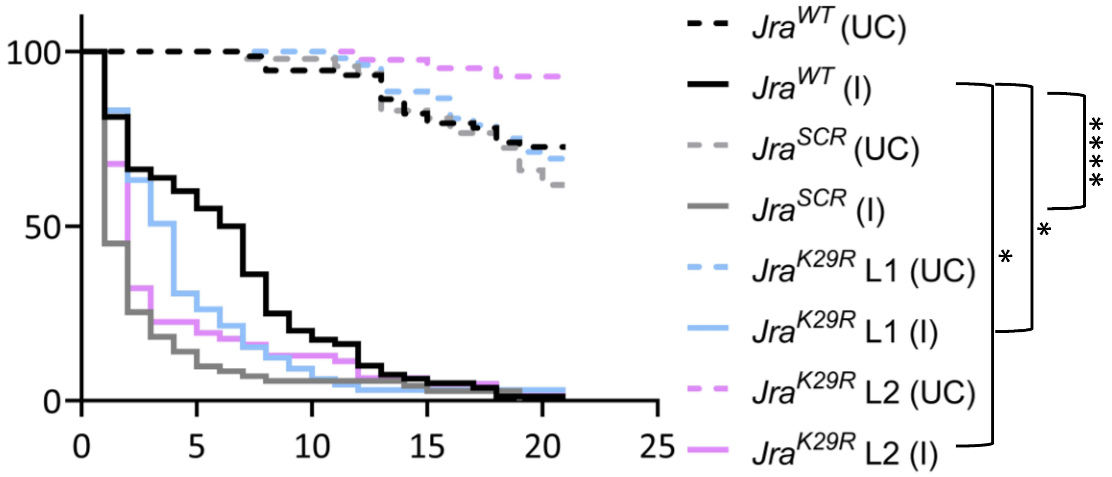

C

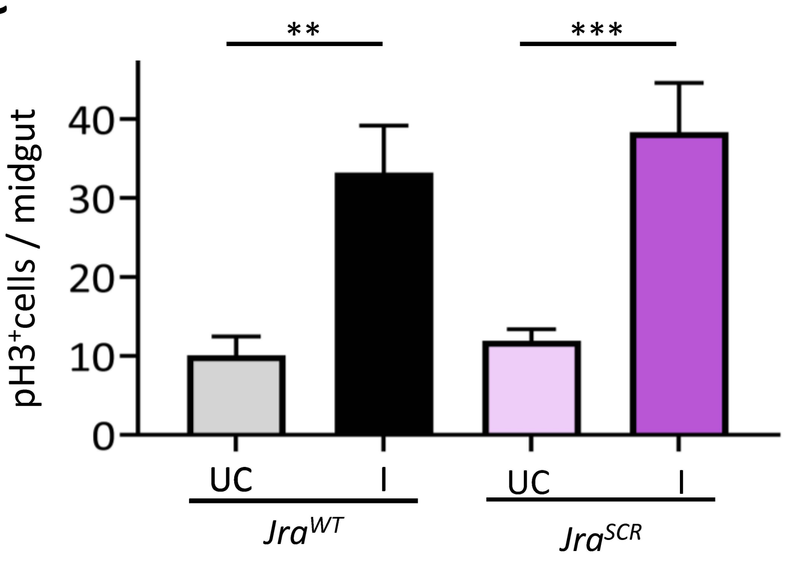

Figure S10: Jra^{SCR} dampens the gut immune response.

A. Tabular representation of the total number of flies used for experiments and number of independent experiments performed in Fig 5.

B. Survival curves of unchallenged (UC, dashed lines) and orally infected (I, closed lines). L1 (blue) and L2 (purple) indicate two independent Jra^{K29R} lines used in the experiment. Number of flies used in the experiments; Jra^{WT} (UC) – 73; Jra^{WT} (I) – 80; Jra^{K29R} L1 (UC) – 52; Jra^{K29R} L1 (I) – 65; Jra^{K29R} L2 (UC) – 42; Jra^{K29R} L2 (I) – 62; Jra^{SCR} (UC) – 47 and Jra^{SCR} (UC) – 71. Log-rank test for trend was used to individually compare Jra^{K29R} (I), Jra^{K29R} *L1* (I) and Jra^{K29R} *L2* (I) to Jra^{WT} (I). ****$p<0.0001$; *$p=0.01$. Data pooled from 2 independent experiments.

C. Quantitation of number of pH3^{+} cells per midgut of UC flies and flies orally fed with *P.e.* **:$p=0.0071$; ***$p=0.0005$ as determined by 2-way ANOVA with Tukey's post-hoc test. Number of gut used; Jra^{WT} (UC) – 20; Jra^{WT} (I) – 23; Jra^{SCR} (UC) – 24; Jra^{SCR} (I) – 24. Means and SEMs represented.
