# Supplementary material for: SUMOylation of Jun fine-tunes the Drosophila gut immune response
Source: PLoS Pathog. 2022 Mar 7;18(3):e1010356. doi: 10.1371/journal.ppat.1010356 (PMC8929699; doi:10.1371/journal.ppat.1010356)
Supplement: S11 Fig — (PDF) [file ppat.1010356.s011.pdf]

Suppl. Figure 11

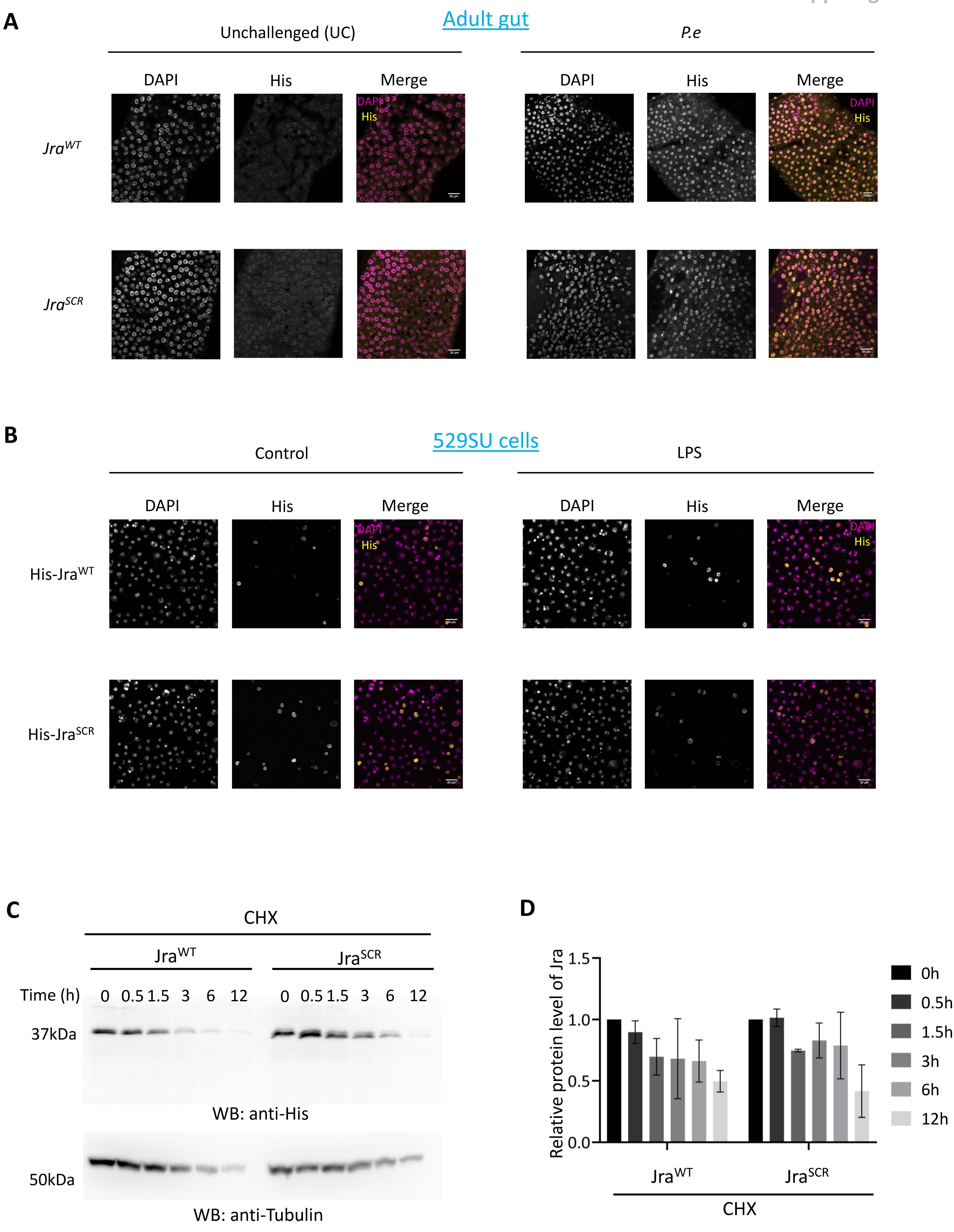

Figure S11: *Jra*^{WT} and *Jra*^{SCR} are similar in terms of expression levels and protein stability.
A. Expression of endogenous Jra in the guts of *Jra*^{WT} and *Jra*^{SCR} without and with *Pe* infection. During infection, there is an increase in Jra staining in both genotypes suggesting an increase on protein levels.
B. Expression of 6XHis tagged Jra (WT and SCR) transiently transfected into 529SU cells without and with LPS induction. Staining shows no change in localization between the genotype in either conditions.
C. Representative WB image of 529SU lysates transiently transfected with 6XHis tagged Jra^{WT} and 6XHis tagged Jra^{SCR} treated with Cycloheximide (CHX) to inhibit protein synthesis and score for the stability of both the variants.
D. Quantitation of relative levels of Jra (normalised to Tubulin) of the Cycloheximide experiment. Two-way Anova with Tukey post-hoc test was performed to compare different conditions. Data pooled from three independent experiments.
