# Supplementary material for: SUMOylation of Jun fine-tunes the Drosophila gut immune response
Source: PLoS Pathog. 2022 Mar 7;18(3):e1010356. doi: 10.1371/journal.ppat.1010356 (PMC8929699; doi:10.1371/journal.ppat.1010356)
Supplement: S12 Fig — (PDF) [file ppat.1010356.s012.pdf]

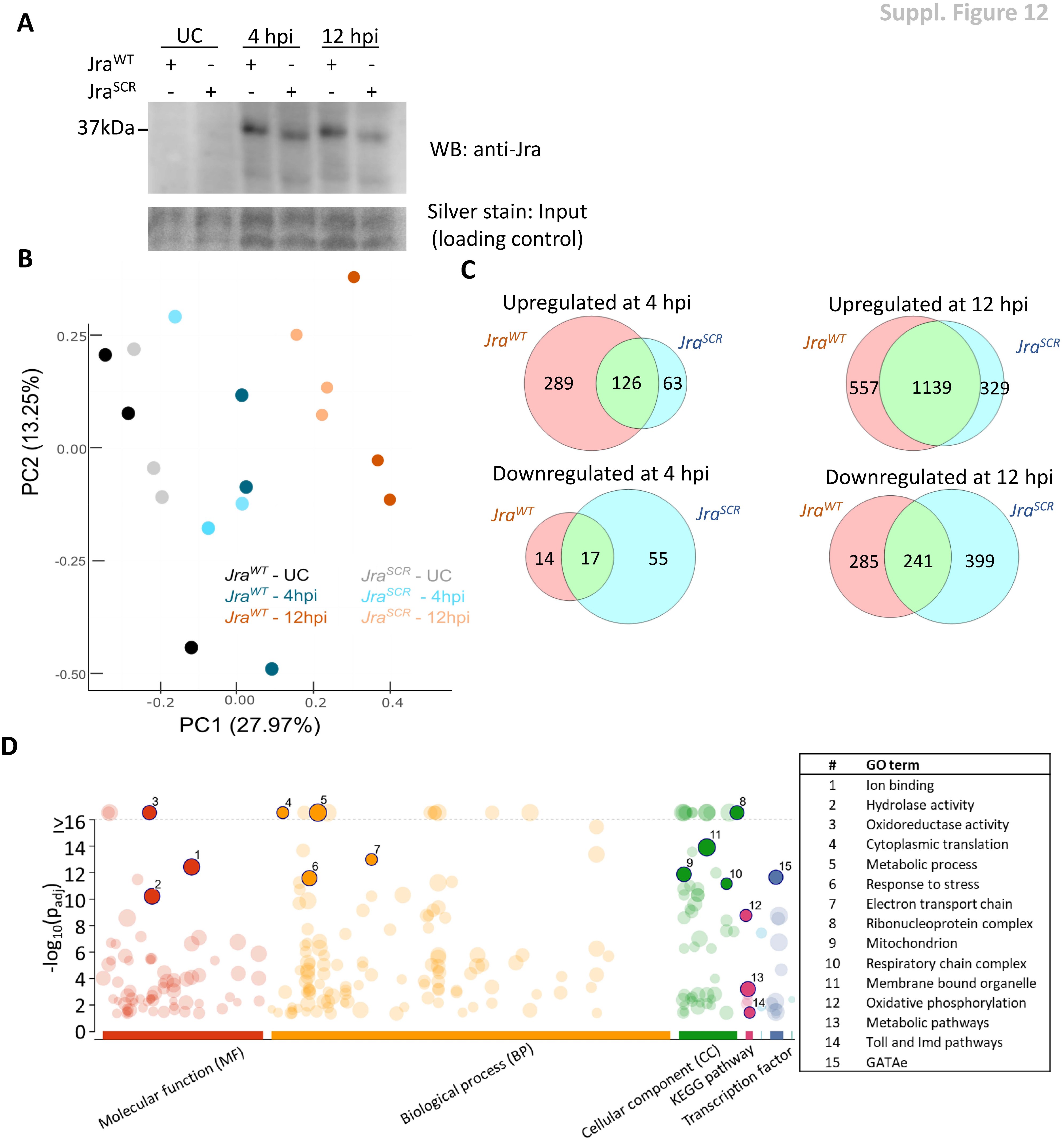

#	GO term
1	Ion binding
2	Hydrolase activity
3	Oxidoreductase activity
4	Cytoplasmic translation
5	Metabolic process
6	Response to stress
7	Electron transport chain
8	Ribonucleoprotein complex
9	Mitochondrion
10	Respiratory chain complex
11	Membrane bound organelle
12	Oxidative phosphorylation
13	Metabolic pathways
14	Toll and Imd pathways
15	GATAe

Figure S12: Gut transcriptomics of *Jra^{WT}* and *Jra^{SCR}*.

A. Western blot of lysates from the gut tissue (n=60) without (UC) and with oral infection (4hpi and 12hpi) of *Jra^{WT}* and *Jra^{SCR}* flies. Jra shows little or no expression in the guts without an immune challenge. Upon infection, the expression of Jra increases and the levels are comparable between the two genotypes. 25% of the same samples were loaded on to an acrylamide gel and silver staining was performed to show equal loading of the lysates.

B. Principal component analysis (PCA) plot showing the first two principal components of the entire data set.

C. Venn diagram of significantly differentially expressed genes (FDR<0.1) upregulated and downregulated in *Jra^{WT}* and *Jra^{SCR}*. The intersection shown in a shade of green represent genes that are common in *Jra^{WT}* and *Jra^{SCR}*.

D. Gene ontology (GO) enrichment analysis of the significantly differentially expressed genes from the entire data set. Some key GO terms are highlighted and described
