# Supplementary material for: SUMOylation of Jun fine-tunes the Drosophila gut immune response
Source: PLoS Pathog. 2022 Mar 7;18(3):e1010356. doi: 10.1371/journal.ppat.1010356 (PMC8929699; doi:10.1371/journal.ppat.1010356)
Supplement: S14 Fig — (PDF) [file ppat.1010356.s014.pdf]

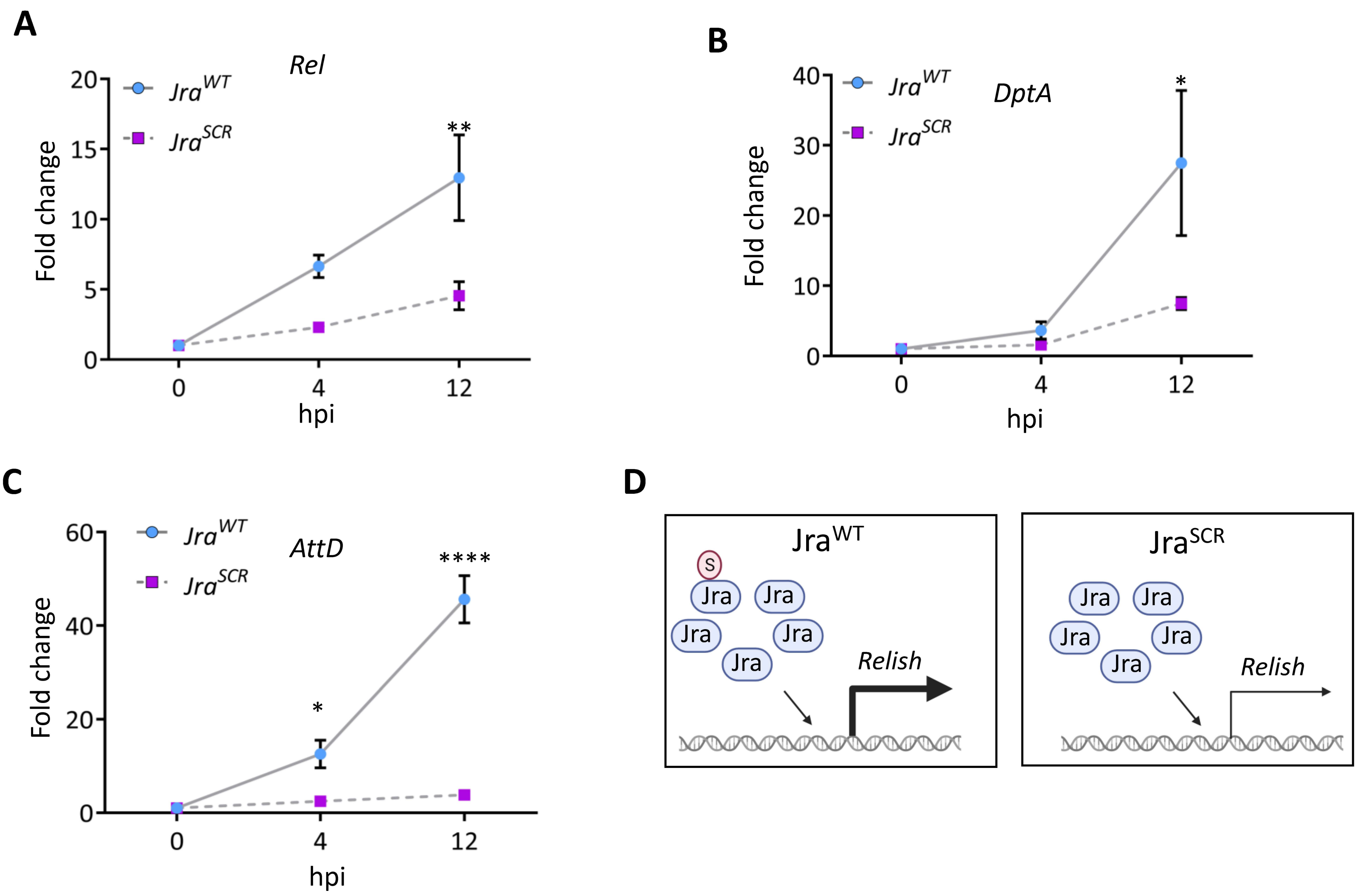

Figure S14: *Jra*^{SCR} shows insufficient activation of *Rel*.

A-C. qRT-PCR data showing the activation of *Rel* (**A**), *DptA* (**B**) and *AttD* (**C**) transcripts during gut infection in *Jra*^{WT} and *Jra*^{SCR}. *p=0.0182 (*DptA*-12hpi), *p=0.0365 (*AttD*-4hpi), **p=0.0027, and ****;p<0.0001 as determined by 2-way ANOVA with Bonferroni's post-hoc test for multiple comparisons. Data from three independent experiments. Means and SEMs represented.

D. Schematic representation of the effect of SUMOylation of Jra on *Rel* transcription during infection. In the left panel, Jra^{WT} can be conjugated to SUMO, and this feature helps in the normal activation of *Rel* during the immune response. Whereas, in the case of Jra^{SCR} (right panel), which cannot conjugate to SUMO, *Rel* expression is significantly weaker.
