# Supplementary material for: SUMOylation of Jun fine-tunes the Drosophila gut immune response
Source: PLoS Pathog. 2022 Mar 7;18(3):e1010356. doi: 10.1371/journal.ppat.1010356 (PMC8929699; doi:10.1371/journal.ppat.1010356)
Supplement: S15 Fig — (PDF) [file ppat.1010356.s015.pdf]

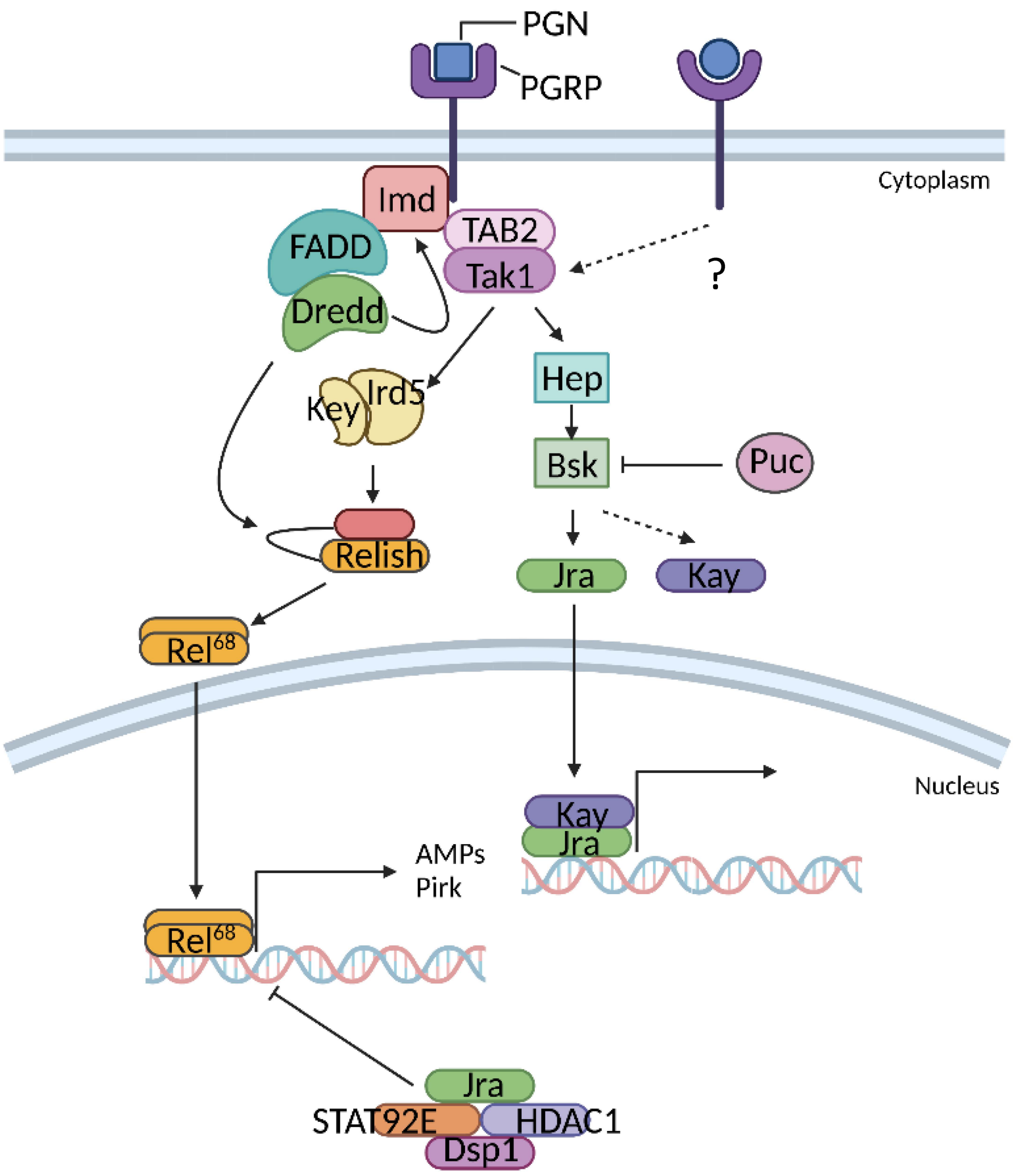

Figure S15: The Imd/JNK pathway in the *Drosophila* immune response.
Upon binding of peptidoglycan (PGN) molecules to the peptidoglycan recognising proteins (PGRPs) presents on the surface of the cell, the Imd pathway gets activated. The adaptor molecule, Imd is cleaved and recruits the TAB2/Tak1 complex. This phosphorylates and activates the IKK complex comprising Key and Ird5. The IKK phosphorylates and activates Rel. Active Rel is cleaved by Dredd and translocates into the nucleus to activate the transcription of antimicrobial peptides (AMPs) and other immune factors. Tak1 parallelly phosphorylates and activates the JNKK, Hemipterous (Hep). Hep activates the JNK, Basket (Bsk) and this inturn activates the AP-1 complex consisting of the dimer Jra and Kay. Jra interacts with HDAC1, STAT92E and Dsp1 to antagonise the activation of *AttA* by Rel.
