# Supplementary material for: SUMOylation of Jun fine-tunes the Drosophila gut immune response
Source: PLoS Pathog. 2022 Mar 7;18(3):e1010356. doi: 10.1371/journal.ppat.1010356 (PMC8929699; doi:10.1371/journal.ppat.1010356)
Supplement: Attachment — Submitted filename: Jra-Response-reviewers.pdf [file ppat.1010356.s019.pdf]

Response to Reviewers

Reviewer #1

Part I: Summary

Please use this section to discuss strengths/weaknesses of study, novelty/significance, general execution and scholarship.

Reviewer #1: In this manuscript, Soory and Ratnaparkhi studied the role of the transcription factor Jra in drosophila immune response. Using a large number of mutant flies, they demonstrated that Jra limits the induction of a protective immune response to pathogens. Then they studied the SUMOylation of Jra. First, they identified the main SUMOylated lysines in vitro and in cellulo and second, they analyzed the effect of their mutation on flies' response to infection and the regulation of gene expression. This manuscript provides interesting new data on the role of SUMOylation in the control of transcription factors activity in a living organism. Nevertheless, except for few genes, the effects on transcription are not always very clear, which questions their biological relevance.

1. We thank the reviewer for their positive comments on the manuscript. As stated by the reviewer, a major novelty of the research is the elucidation of roles for SUMOylation of a transcription factor in a living organism – which sets it apart from the more numerous studies using cells in culture. The use of CRISPR/Cas9 mediated genome editing to create a SUMO conjugation resistant variant of Jra is the cornerstone of this manuscript.

The referee would like us to defend and highlight the biological relevance of the study. Jra is obviously a very important transcription factor in fly immune response, but has not been studied extensively in this context, with less than ten papers in the field over the last two decades. Our study, itself, is the first thorough and detailed study of Jra function in the Gut immune response. Reduction of Jra signalling in gut enterocytes, achieved in our experiments by hypomorphic alleles, leads to protection against *P. entomophila*, clearly suggesting that Jra signalling dictates the ability of the pathogen to infect and kill the host. Our study in fact clarifies the confusion in literature, with our study consistent with previous studies (Kim et al., 2007; Kim et al., 2005) who first demonstrated that Jra (AP1) works to negatively regulate the immune response, but not that of others (Delaney et al., 2006; Silverman et al., 2003).

2. In addition to uncovering the physiological role of Jra in regulating the gut immune response, we report a large network of genes that transcriptionally respond to Jra. We call this regulatory network, the Jra dependant immune gene regulatory network (i-GRN). This list of around 4500 genes include antimicrobial peptides (AMPs), signalling ligands, transcription factors and transcriptional regulators. Using a specific antibody that we have generated against full length Jra, we demonstrate that Jra binds to the promoters of a subset of transcription factors that have been uncovered in our study and thereby could regulate their transcription. Interestingly, a few of these have been previously implicated in regulating the gut immune response. Our study thus directly demonstrates a role for Jra in an infection model with a strong gut phenotype and the underlying molecular mechanism.

3. The next major question is the biological relevance of SUMOylation in Jra function. Again, here, using a SUMO conjugation resistant Jra allele (Jra^{SCR}) generated using CRISPR/Cas9 tecnology, we

demonstrate that the SUMOylation acts to dampen Jra function and this favors the host. *Jra*^{SCR} animals are in fact more sensitive than the wild type allele to *P. entomophila* infection and SUMOylation (or the lack of it, in the Jra^{SCR}) significantly changes the kinetics of the transcriptional response of the gut to the pathogen. Does *P. entomophila* regulate the host SUMO conjugation machinery? Seminal papers in the mammalian field have shown that pathogens such as *Listeria* (Ribet et al., 2010), *Shigella* (Fritah et al., 2014) and *Salmonella* (Verma et al., 2015) capture the host SUMO conjugation machinery to improve their infectivity. *Pe* may use similar molecular mechanisms as a means to modulate host SUMOylation. Studying the SUMOylation of Jra from the perspective of the host which is responding to a pathogen, in an organism, is thus an important biological question.

To summarize, our work supports the idea that reduction of Jra signalling strengthens the immune response, underscoring a role for Jra as a negative regulator (Kim *et. al.* 2005, 2007) in gut immunity, while SUMO conjugation of Jra is antagonistic to its signalling function, acting as a brake for Jra mediated transcription, in line with earlier proposals from mammalian systems (Bossis *et. al.*, 2005; Tempe *et. al*., 2014). Hence we propose that dynamic conjugation/de-conjugation of SUMO of Jra is a crucial step, at the level of Jra target promoters, to evoke a robust immune response in the gut.

Part II – Major Issues: Key Experiments Required for Acceptance
Please use this section to detail the key new experiments or modifications of existing experiments that should be absolutely required to validate study conclusions.

Generally, there should be no more than 3 such required experiments or major modifications for a "Major Revision" recommendation. If more than 3 experiments are necessary to validate the study conclusions, then you are encouraged to recommend "Reject".

Reviewer #1: (No Response)

Part III – Minor Issues: Editorial and Data Presentation Modifications
Please use this section for editorial suggestions as well as relatively minor modifications of existing data that would enhance clarity.

Reviewer #1: Figure 3E: it is quite difficult to compare the two conditions W1118 and JRARNAi and have a clear view of the role of JRA in the expression of the genes. It would be easier to have ratio between the two genotypes in addition to the ratios.
4. We thank the reviewer for this useful suggestion. We have added the information about ratios as an independent column within Fig 3C and Fig 6E.

Figure S6B: here again, the difference between w1118 and JRA-RNAi is not so obvious from the heatmap, although I agree there are some differences. It is important to identify the genes with a Log2FC>1 or <-1 between the two conditions for each time point.
5. The heatmap is generated to provide the reader an overall visual picture of the expression of differentially regulated genes in all the conditions and which are considered direct targets of Jra. Carefully looking at the heatmap, one can appreciate that as the infection progresses, there is a clear change in expression of the genes in *w*^{1118} (compare lanes 1, 3 and, 5 in Fig. S6B) and *Jra*^{RNAi}

(compare 2, 4 and 6 in Fig. S6B). The genes that are significantly differentially expressed have been listed in Tables. S1 and S2.

Figure 4: the informations on the generation of JRAscd could be moved to the method part and the figure to the supplementary data.

6. *Jra*^{SCR} is an important reagent generated in this manuscript. From the year of development of genome editing by CRISPR/Cas9 technology in *Drosophila*, there are only a handful of examples in literature where the genomic sequence was successfully mutated (point mutations) using a knock-in strategy. Moving this crucial aspect into the supplementary data would be, to our mind, grossly undermine the importance and effort put in to generate the mutant fly line. The generation and validation of the Jra$^{K29R,\ K190R}$ mutant is the cornerstone of this manuscript and with us describing it as an active allele of Jra, will be of greater use the fly community

Figure 5A-B: the conclusion of the role of JRA SUMOylation on flies survival upon pe infection is not clear.

7. Our results supports the idea that reduction of Jra signalling strengthens the immune response, underscoring a role for Jra as a negative regulator, as proposed in mammals (Kim *et. al.* 2005, 2007) while SUMO conjugation of Jra is antagonistic to its signalling function, acting as a brake for Jra signalling. We have now structured and improved the discussion (pg. 20-23), adding sub-sections to bring out this central feature of our study.

Figure 2 and 5: It is not clear to which comparisons the pvalues are linked to. There should be brackets between the legends on the side of the survival curves

8. Brackets have been added in Fig 2 and Fig 5 accordingly.

Figure S8: I disagree that the PCA analysis shows that both genotypes form different clusters. The clusters are rather linked to the time of infection than to the genotype.

9**.** We thank the reviewer for making this suggestion. We have modified text accordingly (line. 460).

Global reduction of SUMOylation is protective whilst inhibition of JRA SUMOylation increase sensitibity. The authors should discuss this apparent contradiction

10. This is not really a contradiction. The difference is between SUMO's influence on one target (Jra) vs its overall influence on the immune response. There are number of proteins that are SUMO targets in the animal, even in terms of the immune response (Handu et al., 2015). SUMOylation modulates the activity of each target in a context dependent manner with each target influencing the immune response in an independent manner. The protection that we observe on global SUMO knockdown is a net effect of loss of components of the SUMO machinery on the entire GRN during an immune response.

Reviewer #2

(Part I: Summary

Please use this section to discuss strengths/weaknesses of study, novelty/significance, general execution and scholarship.)

Reviewer #2: In the manuscript entitled "SUMOylation of Jun fine-tunes the Drosophila gut immune response" by Soory and Ratnaparkhi, the authors propose that Jra is a modulator of the immune gene regulatory network induced by Pseudomonas entomophila that is regulated through SUMOylation. They identified K29 and K190 as the SUMOylation sites in Jra. In addition, they carried out transcriptome analysis of a fly line expressing the SUMOylation mutant of Jra and also upon downmodulation of global SUMOylation. Their results indicate that a fly line expressing the Jra SUMOylation mutant is more sensitive than the WT fly line to Pseudomonas infection and suggest that SUMOylation reduces Jra activity. The work has several important problems, including absence of controls, that need to be solved.

We thank the reviewer for the positive comments on the manuscript. As the referee points out, we have successfully demonstrated roles for SUMO conjugation of Jra in the fly gut immune response. The concerns that the reviewer has raised pertain specifically to the initial parts of the study – with suggestions for repeating experiments that demonstrate SUMOylation of Jra and the discovery of the lysines targeted by the SUMO machinery. We have repeated the experiments as suggested and have included the additional controls requested by the reviewer. These are listed in a point to point manner, in detail, in section-II of the rebuttal.

Part II – Major Issues: Key Experiments Required for Acceptance

Please use this section to detail the key new experiments or modifications of existing experiments that should be absolutely required to validate study conclusions.

Reviewer #2: Major concerns:

The authors carried out in vitro SUMOylation assays in order to confirm the SUMOylation of Jra and to identify the SUMOylation sites.

This technique has important problems including lack of controls: the authors express Jra proteins fused to GST. GST proteins contain lysine residues that could work as SUMO acceptors. Therefore, the authors must include a GST protein as a negative control in their assays.

1. The *in-bacto* SUMOylation assay was adapted from (Nie et al., 2009). These authors demonstrate that GST does not get SUMOylated under *in-bacto* conditions (figure attached).

In a similar manner, we also find GST is not SUMO conjugated under our experimental conditions. Our data is incorporated into the manuscript as Fig S1.

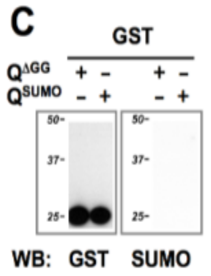

In addition, to probe that the higher molecular bands detected in the Western-blot using anti-His antibody correspond to Jra SUMOylated proteins they need to demonstrate that these bands are recognized by the anti-GST antibody. Moreover, the authors must show the His-SUMO bands corresponding to both the unconjugated and conjugated His-SUMO protein expressed in the bacteria.

2. These figures have been modified according to the reviewer's suggestions with Fig. 1 revamped and changed accordingly. To summarize, we do see the SUMOylated Jra species in all GST western blots (for example right hand panel of Fig. 1A; bottom panel of Fig 1D; Both panels of Fig. 1E).

The SUMOylation pattern shown in fig 1E differ from the one shown in fig.1D (the highest molecular band is missing and an additional lower molecular band is detected in K233). The SUMOylation analysis of Jra-WT must be also included in this panel.

3. We have repeated the experiment and placed the new blots with all variants in a single gel (Fig. 1D), allowing comparison of the Jra^{WT} with all other mutants in a single panel. As shown in the earlier versions of the blots, we do not see any change in the SUMOylation pattern in any of the mutants except K29R and K190R. The data is compiled in Fig 1D.

Similar problems are found in the SUMOylation assay in cells. The band corresponding to Jra SUMOylated protein must be detected with anti-Histidine antibody. In addition, the WB with anti-Flag antibody must show both the conjugated and unconjugated SUMO expressed in the cells.

4. In an ideal scenario where a protein is abundantly SUMOyalated *in-vivo*, one can observe the SUMOylated species by performing a WB post an IP/PD. However, this is not the case in our study as our data suggests that Jra is sparsely SUMOylated in cells. Also, the antibodies used in this study (anti-His and anti-Flag) are both raised in mouse, and this restricts us from stripping the blots and reprobing. Hence, we split the eluate post PD and used the majority (80-90%) of it to probe for Flag that detects SUMOylated Jra and the remaining (10-20%) to probe for His that detects non-SUMOylated Jra. We show the conjugated Jra species (*) in an overexposed blot in Fig. 1F (left panel) while Fig. 1F (right panel) shows the unconjugated SUMO.

Analysis of the SUMOylation of Jra mutants in cells reveals that the Jra-K29R was not SUMOylated, suggesting that mutation of this residue is enough to avoid SUMOylation in cells. Why the results of both SUMOylation assays are different? Some of the fly lines obtained by the authors contain only this mutation. The authors must analyze those fly lines in order to evaluate potential differences with the double mutant.

5. We have generated and tested two independent lines of Jra^{K29R} and performed the survival experiment with appropriate controls (Fig. S9B). We observed that Jra^{K29R} flies do show significant reduction in survival post infection as compared to Jra^{WT}. However, this reduction in lifespan is not as drastic as Jra^{SCR}. This suggests that the degree of SUMOylation dictates the activity of Jra with Jra^{SCR} being the most active and Jra^{K29R} being mildly active.

The authors speculate about the protein expression and SUMOylation regulation upon infection but they do not show any experiment demonstrating their suggestions. They must evaluate:

i) whether infection modulates Jra SUMOylation in cells and, ideally, in flies

6. Infection does modulate Jra SUMOylation in cells. Our current study is based on a quantitative mass spectrometric experiment carried out earlier by our laboratory (Handu et. al., 2015) to detect protein's SUMOylated in response to infection. Crude LPS was used to activate the major signalling pathways in S2 cells. The appended graph (Amar Soory, Ph.D. thesis, 2021) shows a group of proteins which we identified (LPS/control rato's > 2), as potential SUMO targets, with Jra being one such target. In the gut, or even in whole flies, this experiment is challenging simply because we

estimate that Jra expression is low and IPs have high levels of background. SUMOylated species are not detectable in our hands under these challenging conditions.

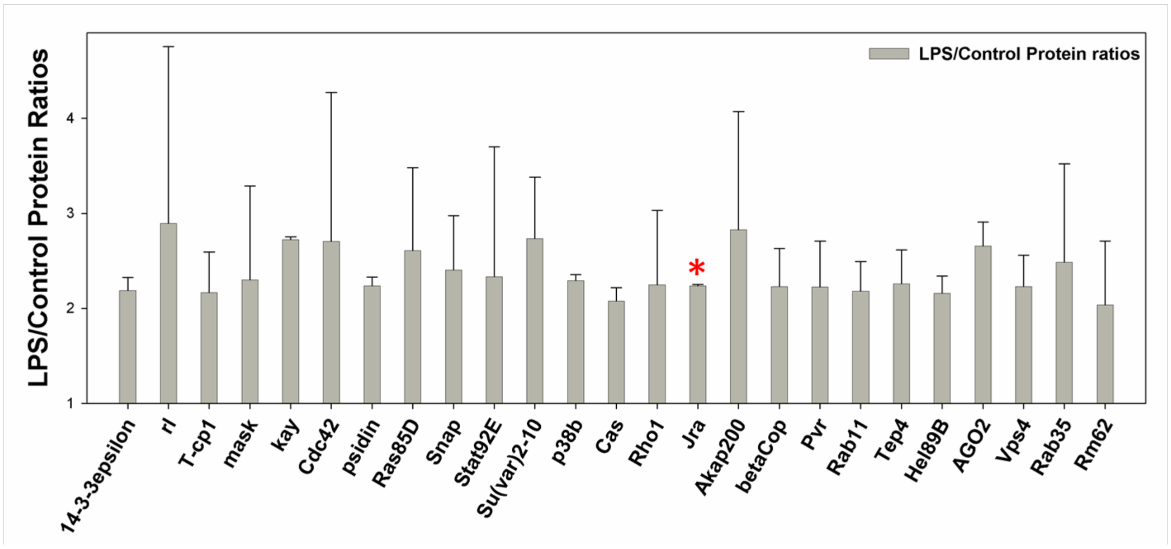

ii) consequences of Jra SUMOylation on protein stability and subcellular localization in cells

7. We have now performed both the suggested experiments in cells and do not observe any change in protein stability or the subcellular localization (Fig. S11B) of Jra in *Jra*^{WT} and *Jra*^{SCR}. These data are added to the manuscript as Fig. S11 C & D. Additionally, we have generated an antibody against Jra and visualized the subcellular localization of Jra in the guts of *Jra*^{WT} and *Jra*^{SCR} (Fig. S11A) and observe no change with and without infection.

iii) analyze whether the changes in mRNA levels at different times after infection occur in parallel to its modulation at protein level and of this with the SUMOylation levels.

8. We examined the levels of Jra in the guts of *Jra*^{WT} and *Jra*^{SCR} flies at UC, 4hpi and 12hpi (Fig. S12A). We did not detect Jra in the UC conditions in both the genotypes. As infection progressed, there was an increase in the protein levels of Jra (Fig. S12A). This was in parallel to the modulation of its mRNA levels.

Additional questions

Does BSK modulate the SUMOylation of Jra?

9. This is an interesting question raised by the reviewer. There are certain examples in the literature where SUMOylation talks to other PTMs and one of the well-studied crosstalk is with phosphorylation. In fact, there exists a phosphorylation-dependent SUMO consensus motif (PDSM; ψKX(D/E)XXSP). The phosphorylation of serine increases the SUMOylation in most documented examples (reviewed in (Celen and Sahin, 2020). In case of Jra, K29 and K190 do not belong to a PDSM and might not be influenced by a phosphorylation site in the vicinity.

The SUMOylation on K29 and K190 can always be influenced by a phosphorylation site that is elsewhere in the polypeptide. The SUMOylation of mammalian c-Jun is influenced by the phosphorylation sites present on the N-terminus (Muller et al., 2000) where loss of these phosphorylation sites increases SUMOylation. More often than not, these N-terminal serine and threonine sites are phosphorylated by the JNK kinases. The *Drosophila* JNK, Basket also phosphorylates Jra on the N-terminus. Since we see many similarities between c-Jun and Jra in terms

of SUMOylation and how it influences the activity, it is fair to assume that phosphorylation by Bsk could influence SUMOylation and vice versa. However, we do not have experimental evidence to support this.

Does the activation of JNK in response to other stimuli modulate Jra SUMOylation?
10. Since the main aim of this study was to study the effect of SUMOylation of Jra in the context of immune response, we have not looked at other stimuli like heat stress, oxidative stress or nutrient stress. It would be worthwhile to look at how SUMOylation of Jra influences certain cellular stresses and how activation of JNK modulates Jra SUMOylation during other stimuli. It is difficult to speculate if a common mechanism dictates the outcome of various stimuli or if the outcome is specific to specific stimuli.

Part III – Minor Issues: Editorial and Data Presentation Modifications
Please use this section for editorial suggestions as well as relatively minor modifications of existing data that would enhance clarity.

Reviewer #2: (No Response)

Reviewer #3

(Part I: Summary

Please use this section to discuss strengths/weaknesses of study, novelty/significance, general execution and scholarship.)

Reviewer #3: This manuscript reports how SUMO modification of the transcription factor Jra, the Drosophila Jun ortholog, regulates immune response in the gut upon Pseudomonas entomophila infection. The authors identify K29 and K190 as major SUMO conjugation sites in Jra. Moreover, they show that a decrease in Jra signalling is beneficial in the host response, while expression of the SUMO-deficient JraK29R+K190R mutant is more susceptible to infection with a weakened host response and increased proliferation of Pseudomonas. RNASeq analysis suggests that loss of SUMOylation of Jra alters transcriptional networks of the immune response. Altogether, the data are of potential general interest in the SUMO field and the experiments are for the most part convincing. One strength of this work is the investigation of Jra SUMOylation in an in vivo model.

1. We thank the reviewer for their positive comments on the manuscript. The reviewer appears to be convinced that our data support the idea of SUMO conjugation of Jra regulating transcriptional networks in the gut immune response, a major finding of this study. The reviewer also underscores the novelty of an organismal model of SUMOylation, which is rare in the field. To the best of our knowledge, an experiment using a CRISPR/Cas9 mediated genome edited mutant, which is SUMO conjugation resistant, has not been described in *Drosophila*.

Our point by point response to the concerns raised by the reviewer is as below.

Part II – Major Issues: Key Experiments Required for Acceptance

Please use this section to detail the key new experiments or modifications of existing experiments that should be <u>absolutely</u> required to validate study conclusions.

Reviewer #3: A major concern is that the data are entirely based on the use of the SUMO conjugation resistant JraK29R+K190R mutant. Formally, it cannot be excluded that this mutant lacks other PTMs, such as ubiquitylation or acetylation at the respective lysine residues. This issue could be easily addressed by changing the glutamate residues rather than the lysine residues within the KxE consensus residues around K29 and 190.

2. Though we cannot completely rule out the possibility of lysines 29 and 190 being sites for other post-translational modifications, there is no evidence for such a modification. In literature, acetylation has been studied for c-Jun (Vries et. al, 2001, EMBOJ) and the only site identified is K271, in the basic domain, a fair distance away, in terms of linear sequence from Lys190. For Jra, the only site determined experimentally is K112 (Weinert et. al, 2011, *Sc. Signal*; Zhang et. al,, 2013, *BBA*). Jerry Workman's group has in fact demonstrated (Fig1, Zhang et. al,, 2013) that the Jra(K112R) variant is not Acetylated suggesting that K112 is the only (or major) site for acetylation in flies. We have added a statement in the text (lines 146-149) indicating that K29, K190 have not been identified as acetylation targets in literature.

We agree in principle with the suggestion of targeting Glutamic acid in the SUMO conjugation motif. In practise, in literature, it is routine to target lysine residues for mutation and this strategy dominates the SUMO field. We have also found examples (See panel below; (Hendriks

et al., 2014)) where targeting the Glutamic acid is not as effective as that of targeting the conjugating reside (lysine) directly.

Finally, the strength of the study is the *in-vivo* generation of the Jra(K29R, K190R) double mutant, using CRISPR/Cas9 genome editing. The generation of an equivalent Jra(E31A,192A) mutation would take us approximately an year, with another year required for characterization and conducting the genetic/transcriptomics experiments.

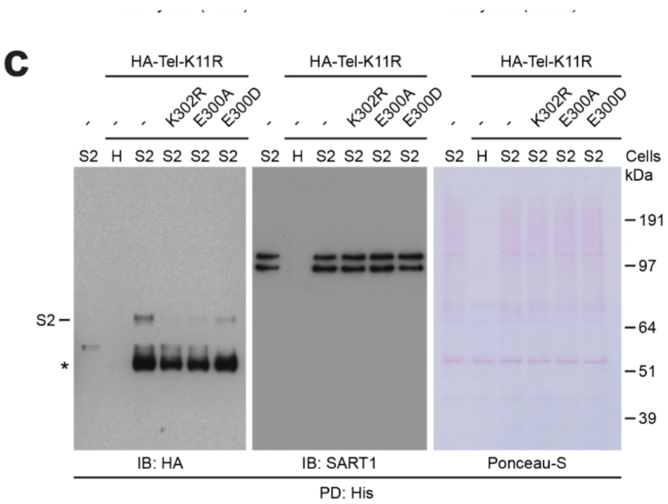

This would likely prevent SUMOylation, but not other PTMs. Furthermore, the work falls short in providing mechanistic insight into the SUMO-controlled JRA activity. I do acknowledge that providing such data, e.g. ChIP or interaction proteomics data, might be difficult in an in vivo setting. However, the authors should at least exploit their cell culture system to compare promoter binding of JraK29R+K190R or wild-type Jra on some of the target genes identifies in the in vivo model.

3. We fully agree with the reviewer that such an experiment using gut tissue is challenging, especially considering the miniscule amount of protein extracted from even large numbers of dissected gut tissue. In early 2021, we had initiated the process of generating an antibody against full length Jra. We were in the process of validating and characterizing his antibody when the manuscript was submitted to *PLOS Pathogens*. As suggested by the reviewer, we could use our cell culture system, in conjunction with the anti-Jra antibody to successfully validate the binding of Jra to the promoters of target genes identified in the *in-vivo* model. We could do the experiment with and without an immune challenge, using the same S2 cell line (529SU) we had used earlier (Figure 1) to demonstrate SUMOylation of Jra. Unlike many S2 cell lines available from the DGRC, the Imd pathway in 529SU cells is sensitive to peptidoglycan contaminants in crude LPS (Kaneko et. al., 2004, Immunity) and gives a robust immune response (Bhaskar et. al., 2000). Importantly, the experiment was done without overexpressing Jra, by immunoprecipitating the cellular Jra natively present on promoters of genes with and without infection. We do not believe that an experiment with overexpressed Jra, either wild-type or mutant would be particularly informative, especially considering that for the mutant overexpression, endogenous wildtype Jra would be present in the cell and that overexpression would bring in artefacts that we have specifically avoided using CRIPSR/Cas9 genome edited lines.

Part III – Minor Issues: Editorial and Data Presentation Modifications
Please use this section for editorial suggestions as well as relatively minor modifications of existing data that would enhance clarity.

Reviewer #3: (No Response)

References (Response to reviewers)

Celen, A.B., and Sahin, U. (2020). Sumoylation on its 25th anniversary: mechanisms, pathology, and emerging concepts. FEBS J *287*, 3110-3140.

Delaney, J.R., Stoven, S., Uvell, H., Anderson, K.V., Engstrom, Y., and Mlodzik, M. (2006). Cooperative control of Drosophila immune responses by the JNK and NF-kappaB signaling pathways. EMBO J *25*, 3068-3077.

Fritah, S., Lhocine, N., Golebiowski, F., Mounier, J., Andrieux, A., Jouvion, G., Hay, R.T., Sansonetti, P., and Dejean, A. (2014). Sumoylation controls host anti-bacterial response to the gut invasive pathogen Shigella flexneri. EMBO Rep *15*, 965-972.

Handu, M., Kaduskar, B., Ravindranathan, R., Soory, A., Giri, R., Elango, V.B., Gowda, H., and Ratnaparkhi, G.S. (2015). SUMO-Enriched Proteome for Drosophila Innate Immune Response. G3 (Bethesda) *5*, 2137-2154.

Hendriks, I.A., D'Souza, R.C., Yang, B., Verlaan-de Vries, M., Mann, M., and Vertegaal, A.C. (2014). Uncovering global SUMOylation signaling networks in a site-specific manner. Nat Struct Mol Biol *21*, 927-936.

Kim, L.K., Choi, U.Y., Cho, H.S., Lee, J.S., Lee, W.B., Kim, J., Jeong, K., Shim, J., Kim-Ha, J., and Kim, Y.J. (2007). Down-regulation of NF-kappaB target genes by the AP-1 and STAT complex during the innate immune response in Drosophila. PLoS Biol *5*, e238.

Kim, T., Yoon, J., Cho, H., Lee, W.B., Kim, J., Song, Y.H., Kim, S.N., Yoon, J.H., Kim-Ha, J., and Kim, Y.J. (2005). Downregulation of lipopolysaccharide response in Drosophila by negative crosstalk between the AP1 and NF-kappaB signaling modules. Nat Immunol *6*, 211-218.

Muller, S., Berger, M., Lehembre, F., Seeler, J.S., Haupt, Y., and Dejean, A. (2000). c-Jun and p53 activity is modulated by SUMO-1 modification. J Biol Chem *275*, 13321-13329.

Nie, M., Xie, Y., Loo, J.A., and Courey, A.J. (2009). Genetic and proteomic evidence for roles of Drosophila SUMO in cell cycle control, Ras signaling, and early pattern formation. PLoS One *4*, e5905.

Ribet, D., Hamon, M., Gouin, E., Nahori, M.A., Impens, F., Neyret-Kahn, H., Gevaert, K., Vandekerckhove, J., Dejean, A., and Cossart, P. (2010). Listeria monocytogenes impairs SUMOylation for efficient infection. Nature *464*, 1192-1195.

Silverman, N., Zhou, R., Erlich, R.L., Hunter, M., Bernstein, E., Schneider, D., and Maniatis, T. (2003). Immune activation of NF-kappaB and JNK requires Drosophila TAK1. J Biol Chem *278*, 48928-48934.

Verma, S., Mohapatra, G., Ahmad, S.M., Rana, S., Jain, S., Khalsa, J.K., and Srikanth, C.V. (2015). Salmonella Engages Host MicroRNAs To Modulate SUMOylation: a New Arsenal for Intracellular Survival. Mol Cell Biol *35*, 2932-2946.
